# PanomiR: a systems biology framework for analysis of multi-pathway targeting by miRNAs

**DOI:** 10.1093/bib/bbad418

**Published:** 2023-11-20

**Authors:** Pourya Naderi Yeganeh, Yue Y Teo, Dimitra Karagkouni, Yered Pita-Juárez, Sarah L Morgan, Frank J Slack, Ioannis S Vlachos, Winston A Hide

**Affiliations:** Harvard Medical School, Boston, MA, USA; Department of Pathology, Beth Israel Deaconess Medical Center, Boston, MA, USA; Harvard Medical School Initiative for RNA Medicine, Boston, MA, USA; National University of Singapore, Singapore; École Polytechnique Fédérale de Lausanne (EPFL), Lausanne, Switzerland; Harvard Medical School, Boston, MA, USA; Department of Pathology, Beth Israel Deaconess Medical Center, Boston, MA, USA; Harvard Medical School Initiative for RNA Medicine, Boston, MA, USA; Broad Institute of MIT and Harvard, Cambridge, MA, USA; Harvard Medical School, Boston, MA, USA; Department of Pathology, Beth Israel Deaconess Medical Center, Boston, MA, USA; Harvard Medical School Initiative for RNA Medicine, Boston, MA, USA; Broad Institute of MIT and Harvard, Cambridge, MA, USA; Harvard Medical School, Boston, MA, USA; Centre for Neuroscience, Surgery and Trauma, Blizard Institute, Queen Mary University of London, London E1 2AT, UK; Harvard Medical School, Boston, MA, USA; Department of Pathology, Beth Israel Deaconess Medical Center, Boston, MA, USA; Harvard Medical School Initiative for RNA Medicine, Boston, MA, USA; Harvard Medical School, Boston, MA, USA; Department of Pathology, Beth Israel Deaconess Medical Center, Boston, MA, USA; Harvard Medical School Initiative for RNA Medicine, Boston, MA, USA; Broad Institute of MIT and Harvard, Cambridge, MA, USA; Harvard Medical School, Boston, MA, USA; Department of Pathology, Beth Israel Deaconess Medical Center, Boston, MA, USA; Harvard Medical School Initiative for RNA Medicine, Boston, MA, USA

**Keywords:** microRNA, miRNA regulation, pathways, biological networks, systems biology, miRNA prioritization, pathway analysis, bioinformatics, computational biology, statistical models

## Abstract

Charting microRNA (miRNA) regulation across pathways is key to characterizing their function. Yet, no method currently exists that can quantify how miRNAs regulate multiple interconnected pathways or prioritize them for their ability to regulate coordinate transcriptional programs. Existing methods primarily infer one-to-one relationships between miRNAs and pathways using differentially expressed genes. We introduce PanomiR, an *in silico* framework for studying the interplay of miRNAs and disease functions. PanomiR integrates gene expression, mRNA–miRNA interactions and known biological pathways to reveal coordinated multi-pathway targeting by miRNAs. PanomiR utilizes pathway-activity profiling approaches, a pathway co-expression network and network clustering algorithms to prioritize miRNAs that target broad-scale transcriptional disease phenotypes. It directly resolves differential regulation of pathways, irrespective of their differential gene expression, and captures co-activity to establish functional pathway groupings and the miRNAs that may regulate them. PanomiR uses a systems biology approach to provide broad but precise insights into miRNA-regulated functional programs. It is available at https://bioconductor.org/packages/PanomiR.

## INTRODUCTION

MicroRNAs (miRNAs) are small noncoding RNAs that act as potent regulators of cellular functions and molecular pathways [1]. They posttranscriptionally regulate gene expression and can coordinate gene function across distinct pathways. miRNA dysregulation has been shown to be a central component of the pathogenesis of diverse diseases, including neoplastic conditions and neurodegenerative disorders [2–20]. Because miRNAs can target dozens of genes, characterizing their role in health and disease requires charting of coordinate co-regulation across heterogeneous molecular cascades and pathways. There has been extensive progress in the development of bioinformatic methods for mapping the effects of miRNAs on the regulation of distinct biological pathways [4, 21–27]. However, no framework currently exists for characterizing miRNA regulation across multi-pathway dynamics that drive transcriptional programs in both healthy and diseased states.

Current best practice for the transcriptomic study of miRNA regulation focuses upon miRNA–gene or one-to-one miRNA–pathway relationships. Widely used miRNA–pathway analysis techniques such as gene set enrichment and co-expression analysis primarily detect whether a single pathway is potentially regulated by a miRNA [4, 21]. Enrichment analyses evaluate the presence (overlap) of targets of a single miRNA in a single pathway, aiming to identify pathways with a higher number of targets than expected by chance [21, 22, 28–32]. Many enrichment-based tools rely on precalculated miRNA–pathway relationships and require users to predetermine their pathways of interest from the disease data [23, 27, 33]. Alternatively, correlation methods evaluate the association of the expression of a single miRNA with a gene or a proxy value representing the activity of a pathway [4, 34]. Table 1 describes some of the most widely used methods for miRNA pathway analyses, their scope, implementation and approaches. A one-to-one approach to miRNA–pathway analysis does not match the regulatory landscape of miRNAs. Large-scale functional processes in health and disease coordinate across pathways in multiple ways, including gene sharing, pathway co-activity, multi-pathway co-regulation and cross-talk [35–40]. The shortcoming of current approaches in accounting for these complex relationships and disease-specific expression dynamics limits our ability to detect the potential of a miRNA to regulate highly specific or broadly acting gene expression programs.

**Table 1:** Overview of standard miRNA-pathway analysis methods and PanomiR

Method/Reference	Multi-pathway targeting	Pathway activity dynamics	Pathway coordination/interaction	Open-source software	Tissue-specific customization
PanomiR This work	X	X	X	X	X
miRPath v4.0 [33]	X (Fisher’s method for meta-analysis)				
Wilk and Braun [4]		X		X	X
miRPathDB 2 [23]				X Precalculated (inactive download links)	X Precalculated
MITHrIL [24]		X (via DEG and DE miRNA)		X	X
miRTar [25]				X Web portal non-functional	
BUFET [26]				X	X
miTalos [27]				X Web portal non-functional	X Precalculated

We have developed a framework to address the existing limitations of miRNA–pathway analysis from a systems perspective, to uncover how functional groupings of pathways are coordinated by miRNAs to form gene expression programs. Our system, Pathway networks of miRNA Regulation (PanomiR), discovers central miRNA regulators based upon their ability to target coordinate transcriptional programs. It determines if a miRNA concurrently regulates and targets a coordinate group of disease- or function-associated pathways, as opposed to investigating isolated miRNA–pathway events. PanomiR derives these multi-pathway targeting events using predefined pathways, their dysregulation in disease states, their relative co-activation, gene expression and annotated miRNA–mRNA interactions.

**Figure 1:**
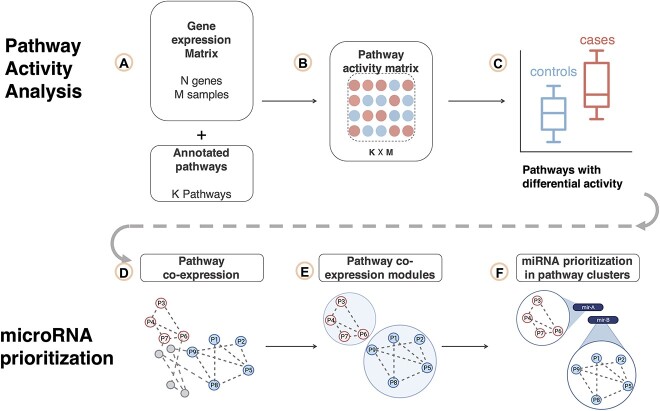
PanomiR workflow. PanomiR prioritizes miRNAs that target coordinate groups of pathways. (**A**) Input gene expression data set and a set of annotated pathways. (**B**) Gene expression data are summarized into pathway activity scores. (**C**) Pathway activity profiles are compared between disease and control subjects to discover differentially regulated pathways. (**D**) Differentially regulated pathways are mapped to the canonical PCxN, where nodes denote pathways and the edges denote correlation of activity scores. (**E**) Within the network of differentially regulated pathways, modules of coordinate pathways are identified using graph clustering algorithms. (**F**) miRNAs are prioritized using annotated miRNA–mRNA interactions (known or predicted) for preferential targeting within each cluster of differentially regulated pathways. The outputs of the pipeline are individual lists of miRNAs with prioritization scores (targeting *P*-values) per each cluster of pathways.

PanomiR profiles the activity of pathways and identifies disease-specific differentially regulated pathways by extending established activity summarization techniques we have previously developed such as Pathprint and Pathway-Drug Network [41–43]. PanomiR identifies network modules of dysregulated pathways, derived from the pathway co-expression network (PcXN.org), to define broad-scale differentially regulated pathway groups [41–43]. PanomiR then determines the miRNAs that target these coordinate pathway groups using a novel statistical test and predetermined miRNA–mRNA interactions [44, 45]. Taken together, these steps produce broad-scale, multi-pathway and disease-specific miRNA regulatory events (Figure 1). PanomiR is available to the community as a free and open-source, user-friendly Bioconductor package.

## MATERIALS AND METHODS

### Overview

**Figure 2:**
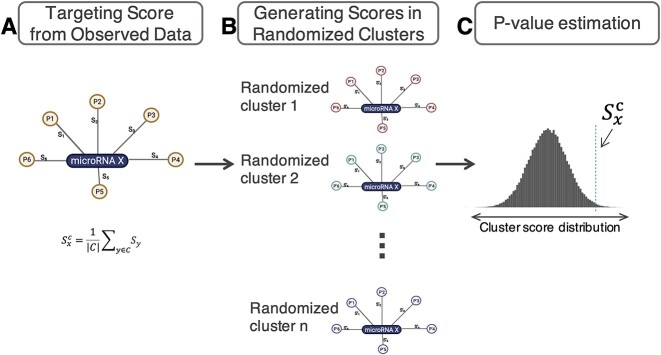
miRNA prioritization from pathway clusters. (**A**) PanomiR generates an observed targeting statistic, *𝑆_𝑥_^c^*, for a miRNA X with respect to C, an observed cluster of pathways. The cluster-targeting statistic is an average individual overlap score for each miRNA-pathway pair. Individual overlap scores (e.g., S1, S2) are functions (inverse normal) of the overlap statistic (Fisher’s exact test) between the miRNA target genes and the pathway member genes (**B**) PanomiR generates an empirical distribution of cluster-targeting scores for a miRNA X by randomly selecting a set of pathways and recalculating the cluster-targeting score. (**C**) The prioritization *P*-value is calculated by comparing the observed targeting statistic, *S_x_^c^*, to the null distribution of the targeting scores of miRNA X. The *P*-value is used to rank the miRNAs.

PanomiR takes as input a user-provided gene expression data set (e.g., RNA-Sequencing (RNA-Seq)) to quantify pathway activity profiles based on annotated pathway databases such as the Molecular Signatures Database (MSigDB) (Figure 1A and B) [46]. Pathway activity profiles are then compared between two conditions (e.g., cancer versus control, wild type versus knockout) to identify and prioritize differentially regulated pathways (Figure 1C). PanomiR constructs a co-activity network of differentially regulated (or disease dysregulated) pathways to determine broad-scale condition-associated groups of functions. It then deconvolves the co-activity network into coherent functional groups using reference pathway co-expression networks, leveraging our previously described pathway activity methods (Figure 1D and E) [41–43]. PanomiR then integrates user-provided miRNA–mRNA interactions (such as predicted targets from TargetScan [44] or experimentally validated interactions from TarBase [45]) to evaluate miRNA regulatory effects on coordinate pathway groups (Figures 1F and 2). The final output of PanomiR is a ranked list of central miRNAs, together with statistical significance levels for each group of differentially regulated pathways, providing an effective means for identification of pathway groups and for key miRNA prioritization, ranking and target detection.

### Capturing pathway activity dynamics

Building upon our previous methodology, Pathprint [41–43], PanomiR ingests a user-provided gene expression data set and calculates pathway activity scores and so captures pathway functional dynamics (Figure 1B). The scores are proxy values for the activity of genes in individual pathways, which, in turn, represent biologically meaningful functional units. By capturing gene expression levels as pathway activity scores, inherent complexity is reduced while tolerance to noise is increased when compared to gene-centric analyses [4, 42, 43, 47]. Pathway activity scores leverage the complex inter-relationships and co-activity of genes. Pathway activity scores examine biological functions in a continuum and detect biological signals where standard differential gene expression analyses fail [4, 34, 41, 43, 47–49].

We capture pathway activity profiles in a two-step process: (a) we rank genes in each sample in descending order, according to their expression, i.e. the highest expressed gene gets the largest rank-score, and (b) we calculate the average squared ranks of genes that belong to a pathway as the activity score. Formally, for a pathway X with n genes, $ {\mathrm{Pathway}}_x=\left\{{g}_1^x,\dots, {g}_n^x\right\}$, the activity score, ${Ac}_{a,x} $, in sample $ a$ is 


$$ {Ac}_{a,x}=\frac{1}{n}{\sum}_i^n{\operatorname{rank}}_a{\left({g}_i^x\right)}^2 $$

where ${\operatorname{rank}}_a\left({g}_i^x\right)$ refers to the rank of gene ${g}_i^x$ (descending order) in sample $a$ based on expression values. We generate activity profiles for each pathway of interest in each sample. Then, pathway profiles are normalized across the input samples (Supplementary Material). PanomiR uses the canonical pathways collection from MSigDB as its pathway database reference [46]. MSigDB is a carefully curated database that represents non-redundant pathways from established pathway repositories such as KEGG and Reactome [46, 50, 51].

### Detection of differentially regulated disease-associated pathways

PanomiR compares pathway activity profiles between case and control subjects to determine functional dynamics in disease. PanomiR defines differentially regulated pathways by determining statistically significant differences in pathway activity profiles between cases and controls using linear models, implemented in the *Limma* package (Figure 1C) [52]. In contrast to enrichment analysis, the linear modeling framework of PanomiR determines the directionality of differential regulation: it defines whether a pathway is upregulated or downregulated in disease subjects (or experimental conditions) and accounts for confounding variables such as batch, sequencing center or any other fixed effects and continuous covariates. PanomiR outputs an ordered table of differentially regulated pathways along with *P*-values of differential regulation, adjusted for multiple hypothesis testing using false discovery rate (FDR) [53].

### Detection of groups of differentially regulated pathways via their co-expression networks

Dysregulation of an individual pathway is rarely an isolated event since pathways share activity and are often co-regulated. PanomiR accounts for co-regulation to place differentially regulated pathways into groups that represent high-level disease programs by exploiting the PCxN [43]: a reference tool that organizes and assesses the shared activity of pathways (Figure 1D). PanomiR leverages PCxN’s network, generated from a curated data set of 3207 expression profiles, providing an independent platform, to query co-activity of all pathways in the MSigDB data set [43, 54].

PanomiR masks PCxN to contain only the subnetwork of differentially regulated pathways that were identified from the two-group data analysis in the previous step. In the masked network, nodes represent differentially regulated pathways and edges activity-correlation of pathways. PanomiR subsequently identifies densely interconnected differentially regulated pathway subnetworks using graph clustering algorithms (Figure 1E). The default clustering algorithm of PanomiR is Louvain, but PanomiR can use other clustering methods that are available in the igraph R-package [55]. The subnetworks denote clusters of highly correlated coordinate groups of differentially regulated pathways driving disease- or condition-specific functions.

### miRNA prioritization within clusters of differentially regulated pathways

PanomiR exploits the concept that a coordinate group of disease-associated pathways has common miRNA regulators. Using annotated miRNA–mRNA interactions and an empirical statistical test (Figure 2), it analyzes clusters of differentially regulated pathways, to define central miRNAs and captures the extent to which the targets of a specific miRNA are present within a group of coordinate pathways. miRNA regulatory events are then identified in three sequential steps (Figure 2): (i) by calculating individual miRNA-pathway overlap scores, (ii) by generalizing miRNA targeting scores to a group of pathways (i.e., a cluster of differentially regulated pathways) and (iii) by estimating the statistical significance of miRNA targeting scores using an empirical approach. The empirical statistical tests are specific to the input data set, for each miRNA and each cluster of differentially regulated pathways.

In the first step, PanomiR derives the overlap scores for individual miRNA-pathway pairs using *P*-values from Fisher’s exact test, capturing overrepresentation of targets of a specific miRNA in each individual pathway. To make analysis disease-, condition-, tissue- or cell-type- specific, PanomiR calculates overlap scores using only the genes expressed in the input experiment. In the second step, an overall targeting score for a given cluster of pathways (Figure 2) is derived. The clusters of pathways are generated in the previous step using PCxN. Formally, for each cluster of differentially regulated pathways, $C$, the targeting score of a miRNA $x$ is


$$ {S}_x^c=\frac{1}{\mid C\mid }{\sum}_{y\in C}{\varPhi}^{-1}\left(1-{P}_{xy}\right) $$

where ${\varPhi}^{-1}$(.) denotes the inverse of the standard normal cumulative distribution function (CDF) and ${P}_{xy}$ denotes the Fisher’s exact test *P*-value of overlap between the targets of miRNA *x* and genes of pathway *y*. The targeting-score, ${S}_x^c$, is related to Stouffer’s method (with equal weights) for *P*-value aggregation. The inverse normal CDF avoids extreme cases in which an miRNA has many targets in one pathway and only a few targets in other pathways in a cluster.

In the third step, the statistical significance of the targeting score ${S}_x^c$ is determined in order to produce cluster-specific lists of miRNAs ranked by targeting *P*-values. The targeting-score does not constitute, by itself, an unbiased measure of miRNA-targeting as it might depend on the number of targets of a miRNA. To create an unbiased measure, PanomiR also derives an empirical-targeting *P*-value, $P({S}_x^c)$, for a score of ${S}_x^c$. This *P*-value denotes the probability of observing a larger-targeting score from a random cluster of pathways (with $\mid C\mid$ members) than the one observed. This empirical probability is derived using a bootstrap sampling approach by selecting randomized groups of pathways and re-calculating their cluster targeting score. This approach directly tackles known or unknown biases in gene annotations for miRNA targets, as have been discussed by our group [21] and others [56, 57]. The output *P*-values are then adjusted for multiple hypothesis comparison using the Benjamini–Hochberg FDR [53].

Given the computational cost of bootstrap sampling, especially to calculate small *P*-values, PanomiR employs a Gaussian approximation approach to estimate $P({S}_x^c)$. In clusters of large-enough size (>30 pathways), ${S}_x^c$ values follow a normal distribution according to the central limit theorem. PanomiR uses pre-calculated Gaussian distribution estimates from 1000 random ${S}_x$values to overcome the computational costs in these cases. In the last step, miRNAs are prioritized based on *P*-values for targeting each cluster. PanomiR’s framework was assessed in a case study of a liver cancer data set from The Cancer Genome Atlas (TCGA) with 368 primary tumor samples and 49 controls (details in Supplementary Material).

### Parameter sensitivity analysis

All steps of PanomiR’s framework (Figure 1) were evaluated across input parameters and algorithmic choices. The sensitivity of PanomiR’s pathway activity profiling (steps 1–3) was evaluated using synthetic data derived from randomized pathways and disease groups (case/control) in comparison with biologically meaningful pathways and disease samples (details in Supplementary Material, Supplementary methods). The consistency of identified pathway clusters from PCxN was evaluated by assessing clustering algorithms and pathway input sizes and compared with Jaccard similarity analysis (steps 4–5). Sensitivity analysis of Gaussian estimated miRNA *P*-values of pathway groups was performed with respect to individual pathways using jackknife estimation (step 6). The sensitivity of miRNA prioritization to parameters of predicted miRNA–mRNA interactions was assessed using Jaccard similarity analysis (step 6). Supplementary material contains details of the implementation and specifications of parameter sensitivity analysis.

### Comparative assessment of PanomiR and established miRNA–pathway analysis tools

PanomiR’s miRNA prioritization was compared with widely used miRNA–pathway analysis tools DIANA-miRPath v4.0, MIENTURNET and MITHRIL (parameter details in Supplementary Material) [22, 24, 33]. PanomiR was also directly compared against Fisher’s exact test (hypergeometric test) because the latter is incorporated in the vast majority of existing tools including DIANA-miRPath, MIENTURNET, miTalos and miRPathDB v2.0 [22, 23, 27, 33]. In addition to packages and webservers, two adaptations of existing miRNA–pathway analysis approaches were implemented and assessed in the PanomiR’s ecosystem: (1) miRNA hits: determining the number of pathways within a given cluster that were significantly enriched for targets of a miRNA and (2) *P*-value aggregation: using Fisher’s method and/or Stouffer’s method to obtain one single *P*-value that combines enrichment *P*-values of a miRNA within all pathways in a cluster. In addition to evaluation of miRNA prioritization, PanomiR’s pathway activity profiles were compared to MITHRIL’s results for assessment of disease-associated pathways. PanomiR was compared to other tools in terms of number of miRNAs detected, biological relevance of miRNAs attributed to their respective pathway groups and bias in prioritization of miRNAs with a large number of targeted genes.

## RESULTS

We present a case study of PanomiR’s utility to provide a systematic, unbiased and biologically meaningful determination of regulatory miRNAs. We applied our system (Figure 1) to a liver cancer gene expression data set from The Cancer Genome Atlas (TCGA) comprising 368 primary tumor samples and 49 controls (data preprocessing detail in Supplementary Material, Supplementary methods) [58, 59]. PanomiR identified differentially regulated pathways and uncovered their regulating miRNAs in liver cancer. We assessed the biological relevance of the readouts and evaluated the parameter sensitivity and statistical robustness of PanomiR *in silico*. Our framework recapitulated known central miRNAs in hepatocellular carcinoma with a biologically meaningful assignment of pathways under their regulation, unbiased by the number of genes targeted by each miRNAs. By comparing PanomiR’s results with liver cancer literature and other miRNA–pathway analysis tools, we demonstrate its ability to unbiasedly infer informative multi-pathway targeting events by miRNAs.

### PanomiR detects multiple liver cancer-associated pathways

We generated and compared pathway activity profiles from normal tissues adjacent to tumor (TCGA abbreviation: NT, *n* = 49) and primary solid tumors (TCGA abbreviation: TP, *n* = 368) from liver cancer gene expression RNA-Seq data and using the MSigDB pathway database. PanomiR detected 428 upregulated and 397 downregulated pathways in TP compared to NT (FDR < 0.01, total pathways 1220; Tables 2 and S1). The large-scale differences in pathway activity profiles closely mirror the differential expression results at the gene level where more than 50% of the genes were differentially expressed (DE) based on a similar statistical design (FDR < 0.01; *n* = 7801; total genes = 14 212).

**Table 2:** Detection of differentially regulated pathways in liver cancer. Most significant differentially regulated pathways identified by PanomiR according to the *P*-value of differential activity between tumor (TP) and normal tissues (NT) from TCGA database. Differential regulation *P*-values were derived using linear models using the *limma* package by comparing pathway activity profiles of TP versus NT. Differential activation adjusted *P*-values are for multiple hypothesis testing using FDR. Direction denotes upregulation or downregulation of pathway activity in TP versus NT. Enrichment adjusted *P*-values for pathways are provided for comparison. Enrichment *P*-values were derived from the differentially expressed genes (|FC| >1, FDR <0.05). The column ‘#DE genes’ shows the number of differentially expressed genes (TP versus NT) that are present in the pathway

Differentially regulated pathways	Adjusted *P*-value	Direction TP versus NT	Enrichment adj. *P*-value	#DE genes
REACTOME: NUCLEAR SIGNALING BY ERBB4	1.13E−30	DOWN	1	5
KEGG: NEUROACTIVE LIGAND RECEPTOR INTERACTION	1.13E−30	DOWN	0.00109	27
KEGG: JAK STAT SIGNALING PATHWAY	3.19E−28	DOWN	1	12
REACTOME: CLASS A1 RHODOPSIN LIKE RECEPTORS	2.98E−27	DOWN	0.0114	28
REACTOME: GPCR LIGAND BINDING	4.2E−27	DOWN	0.0265	35
REACTOME: HDL MEDIATED LIPID TRANSPORT	7.57E−26	DOWN	0.53	4
PID: TCR CALCIUM PATHWAY	1.29E−25	DOWN	1	3
BIOCARTA: GATA3 PATHWAY	1.25E−24	DOWN	1	1
REACTOME: ASSEMBLY OF THE PRE REPLICATIVE COMPLEX	2.19E−24	UP	0.849	11
REACTOME: ORC1 REMOVAL FROM CHROMATIN	1.03E−23	UP	1	9
KEGG: TRYPTOPHAN METABOLISM	1.37E−23	DOWN	0.000547	15
BIOCARTA: ACTINY PATHWAY	4.77E−23	UP	1	0
REACTOME: PROTEIN FOLDING	5.59E−23	UP	1	0
KEGG: CYTOKINE–CYTOKINE RECEPTOR INTERACTION	1.71E−22	DOWN	0.0543	33
PID: ARF6 PATHWAY	2.55E−22	DOWN	1	5
BIOCARTA: IL1R PATHWAY	3.87E−22	DOWN	1	3
REACTOME: REGULATION OF INSULIN LIKE GROWTH FACTOR IGF ACTIVITY BY INSULIN LIKE GROWTH FACTOR BINDING PROTEINS IGFBPS	5.02E−22	DOWN	0.0474	6
REACTOME: SIGNALING BY GPCR	1.28E−21	DOWN	0.447	52
REACTOME: PREFOLDIN MEDIATED TRANSFER OF SUBSTRATE TO CCT TRIC	2.63E−21	UP	1	0
REACTOME: LIPOPROTEIN METABOLISM	2.64E−21	DOWN	0.0521	9
PID: IL1 PATHWAY	3.2E−21	DOWN	1	3
REACTOME: M G1 TRANSITION	3.92E−21	UP	0.447	15
BIOCARTA: TOLL PATHWAY	4.12E−21	DOWN	1	2
KEGG: UBIQUITIN MEDIATED PROTEOLYSIS	7.43E−21	UP	1	4
REACTOME: MRNA SPLICING MINOR PATHWAY	8.3E−21	UP	1	0

**Figure 3:**
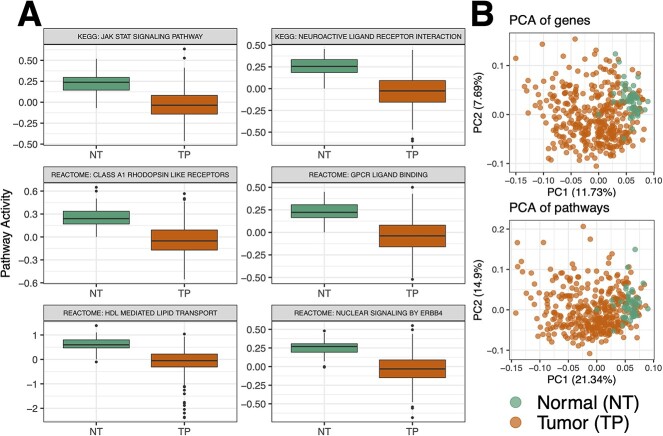
Pathway activity analysis of the Liver Hepatocellular Carcinoma data set from the Cancer Genome Atlas. (**A**) Detection of differentially regulated liver cancer pathways by comparison of pathway activity profiles between normal tissues adjacent to tumors (NT) and tumor primary (TP) samples from TCGA data. Boxplots show the most significant differentially regulated pathways selected based on *P*-values of difference between NT and TP (Table 2). (**B**) Principal component analysis (PCA) projection of the samples based on either all genes or all pathways. Pathway summarization in PanomiR allows to analyze the activity of pathways in a continuum. PCA of pathways conserves sample groups and captures a higher variation compared to the PCA of genes.

Differentially regulated pathways reflected well-established dysregulated functions in liver cancer (Table 2). For example, NUCLEAR SIGNALING BY ERBB4 was downregulated in TP and activated in NT and has the highest statistical significance among all pathways (Figure 3A, Table 2). Downregulation of ERBB4 in tumors is in concordance with a well-established body of evidence on the roles of ERBB signaling as a tumor suppressor in liver cancer [60, 61]. In addition, we found downregulation of HDL-MEDIATED LIPID TRANSPORT in tumor tissues, corroborated by several reports on lipid disorders in liver cancer including decreased plasma levels of HDL [62, 63]. These results suggest the utility of PanomiR in detecting differentially regulated disease functions through pathway activity analysis.

We compared the pathway readouts of PanomiR with pathway enrichment analysis of DE genes from the same data set. Enrichment analysis (Fisher’s exact test) identified 51 enriched pathways (FDR < 0.01, Table S2) within the DE genes (differential gene expression: FDR < 0.05; |LogFC| > 1; Supplementary material). Of these enriched pathways, 50 were also determined as differentially regulated by PanomiR. Significant overlap between the results suggests that PanomiR recapitulates the majority of enrichment analysis readouts (Fisher’s exact test *P*-value = 3.5 × 10^−8^). PanomiR detected liver cancer pathways that were missed by enrichment analysis (Tables 2 and S1). For example, the top liver cancer-associated pathway according to PanomiR, NUCLEAR SIGNALING BY ERBB4*,* was not detected by enrichment analysis (*P*-value = 1). Enrichment analysis (overrepresentation test) prioritizes pathways with more DE genes than expected by chance. This means that enrichment analysis misses pathways with differential activity between disease and controls subjects that do not have increased DE gene proportions. These results highlight two main advantages of PanomiR over pathway enrichment analysis: (a) the ability to detect significant functional dysregulation in disease even in absence of significant differential gene expression and (b) the ability to determine the direction of differential pathway regulation, i.e. upregulation or downregulation.

### Synthetic data analysis shows PanomiR captures biologically meaningful signals

To assess the recapitulation of biological signals by PanomiR, we employed two randomization tests (Supplementary Material, Supplementary methods). First, we asked to what extent PanomiR detected differentially regulated pathways in a random assignment of samples to case and control groups in liver cancer (i.e. biologically meaningless classes). PanomiR found a very small number of differentially regulated pathways (mean = 0.054, SD = 1.2) via randomized case/control sample assignment (Table S3). This means that PanomiR is not prone to detect spurious findings (i.e., differentially regulated pathways) in absence of a biological signal.

Next, we examined if using biologically meaningful pathways (as annotated in the MSigDB) demonstrated any advantages over using randomly assigned gene sets. We generated randomized pathways by permuting gene labels to conserve the pathway overlap structure of the original MSigDB data set. We found that annotated gene sets generate a significantly larger number of differentially regulated pathways than randomized ones (one-sided *z*-test *P*-value <3.34 × 10^−5^; mean = 693.785 pathways at an FDR < 0.01; SD = 39.1). To assess PanomiR’s sensitivity irrespective of *P*-values cut-offs, we also compared the distribution of adjusted *P*-values of differentially regulated pathways between MSigDB and randomized pathway collections. A one-sided Kolmogorov–Smirnov test showed a significant difference between the randomized gene sets and known pathways (*P*-value <2.86 × 10^−18^Table S3, Figure S1). This finding shows that biologically meaningful gene sets were more likely to sensitively capture biological signals.

### Identification of coordinate clusters of differentially regulated pathways

Pathways coordinate and co-regulate through various mechanisms, including shared genes. We used the PCxN—where edges represent precalculated correlations between pathways based on independent gene expression data—to detect coordinate groups of differentially regulated pathways [43]. We mapped the 200 most statistically significant differentially regulated pathways onto the PCxN network. We then performed Louvain clustering to identify coordinate pathway groups among the top 200 pathways.

**Figure 4:**
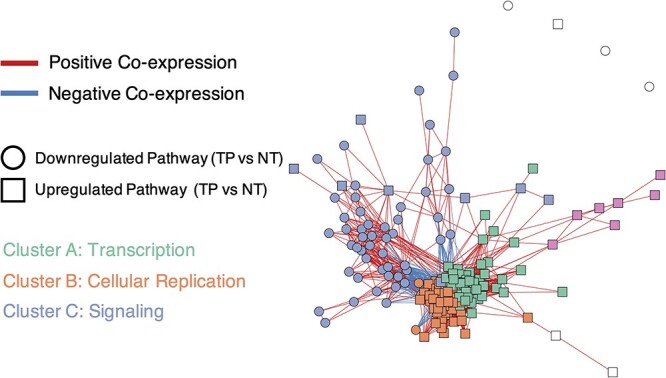
PanomiR deconvolutes coordinate clusters of differentially regulated pathways in liver cancer. The network displays a pathway co-expression map of liver cancer pathways. PanomiR detected three major groups of pathways, defined by the direction of differential regulation and clusters of coexpression. The three classes are (i) activation of transcription in tumors (cluster A); (ii) activation of cellular replication (cluster B); and (iii) deactivation of specific signaling pathways (cluster C). Each node in the network represents a differentially regulated pathway (Table 2). Edges represent canonical coexpression between two pathways, obtained from an independent compendium of gene expression data, derived from the PCxN method. Node colors represent unsupervised network clusters found by the Louvain algorithm. Clusters were manually labeled according to the functional consensus of their pathways.

PanomiR identified three major clusters of differentially regulated pathways (Figure 4) with consistent functions: (i) the largest cluster of differentially regulated pathways (cluster A) contained pathways upregulated in cancer such as SPLICEOSOME, PROTEASOME, TRANSLATION, RNA POLL II TRANSCRIPTION and SIGNALING BY WNT (Supplementary Table S4). Wnt signaling activation is a critical mechanism for transformation of precancerous lesions into liver cancer through proliferation [64]. (ii) The second largest cluster (cluster B) contained pathways related to cell cycle and proliferation (Figure 4, Table S4). (iii) The third cluster (cluster C) contained liver cancer-associated signaling pathways that were either down or upregulated in cancer with terms related to ERBB signaling, IL signaling and NOTCH signaling (Table S4). Differentially regulated pathways within clusters A and B showed a coherent direction of differential regulation in cancer (TP) versus normal adjacent tissues (NT), suggesting coordinate multi-pathway dysregulation driving high-order disease functions.

### Detection of regulatory miRNAs that target clusters of differentially regulated pathways

We evaluated whether coordinate clusters of differentially regulated pathways have common miRNA regulators. In our case study, we examined both experimentally supported (TarBase v8.0; >500 K interactions) and predicted miRNA–mRNA interactions (Targetscan v7.2; >113 K interactions) to detect miRNAs that target each cluster of differentially regulated pathways (Tables 3, 4, S5 and S6). Our results showed that PanomiR identified distinct informative miRNAs for each cluster of liver cancer-associated pathways.

**Table 3:** PanomiR prioritizes regulatory miRNAs in liver cancer using experimentally validated interactions. Prioritized miRNAs for each identified pathway cluster, ranked by PanomiR targeting *P*-value (Figure 2). miRNAs are prioritized based on experimentally validated miRNA–mRNA interaction from TarBase V8.0. Enrichment analysis results are provided for comparison. The column ‘#Pathways enriched’ denotes the number of pathways in the cluster with significant (FDR < 0.25) enrichment in the targets of each miRNA, derived using Fisher’s exact test

Cluster A (*n* = 65)			Cluster B (*n* = 60)			Cluster C (*n* = 58)		
miRNA	Hits (#pathways enriched)	PanomiR adjusted *P*-value	miRNA	Hits (#pathways enriched)	PanomiR adjusted *P*-value	miRNA	Hits (#pathways enriched)	PanomiR adjusted *P*-value
hsa-miR-525-3p	1	2.7E−43	hsa-miR-107	40	1.92E−22	hsa-miR-410-3p	1	2.25E−07
hsa-miR-1307-5p	0	1.4E−27	hsa-miR-124-3p	38	2.04E−22	hsa-miR-552-3p	4	0.00057
hsa-miR-302c-5p	0	6.22E−24	hsa-miR-103a-3p	40	3.24E−21	hsa-miR-5187-5p	1	0.00057
hsa-miR-631	5	6.61E−24	hsa-miR-129-2-3p	37	2.31E−17	hsa-miR-612	2	0.00254
hsa-miR-663a	6	1.64E−23	hsa-miR-1-3p	36	2.43E−17	hsa-miR-198	7	0.0142
hsa-miR-595	4	4.03E−23	hsa-miR-23a-5p	3	8.12E−16	hsa-miR-621	2	0.0172
hsa-miR-933	1	1.04E−22	hsa-miR-663a	5	3.29E−15	hsa-miR-199b-5p	4	0.0209
hsa-miR-510-5p	1	1.04E−22	hsa-miR-449b-5p	33	2.26E−14	hsa-miR-204-3p	1	0.0249
hsa-miR-5009-5p	7	2.23E−22	hsa-miR-147a	41	2.26E−14	hsa-miR-4733-5p	1	0.0503
hsa-miR-2682-5p	0	4.65E−22	hsa-miR-193b-3p	35	3.26E−14	hsa-miR-506-3p	2	0.0503
hsa-miR-486-3p	0	1.77E−19	hsa-miR-192-5p	21	6.28E−14	hsa-miR-564	8	0.0535
hsa-miR-936	1	3.06E−19	hsa-miR-548ar-3p	8	1.69E−13	hsa-miR-1291	6	0.0666
hsa-miR-550b-2-5p	2	3.46E−19	hsa-miR-34c-5p	21	2.91E−13	hsa-miR-299-5p	1	0.0737
hsa-miR-23b-3p	38	4.88E−19	hsa-miR-301a-5p	7	2.99E−13	hsa-miR-373-3p	2	0.0737
hsa-miR-214-5p	4	4.23E−18	hsa-miR-214-3p	35	2.99E−13	hsa-miR-335-5p	2	0.0877

**Table 4:** PanomiR prioritizes regulatory miRNAs in liver cancer using predicted interactions. Prioritized miRNAs for each identified pathway cluster, ranked by PanomiR targeting *P*-value (Figure 2). miRNAs are prioritized based on predicted miRNA–mRNA interaction from TargetScan V7.2. The column ‘pathways enriched’ denotes the number of pathways in the cluster with significant (FDR < 0.25) enrichment in the targets of each miRNA, derived using Fisher’s exact test

Cluster A			Cluster B			Cluster C		
miRNA	Hits (#pathways enriched)	PanomiR adjusted *P*-value	miRNA	Hits (#pathways enriched)	PanomiR adjusted *P*-value	miRNA	Hits (#pathways enriched)	PanomiR adjusted *P*-value
hsa-miR-371a-5p	0	1.06E−38	hsa-miR-191-5p	2	2.53E−19	hsa-miR-219a-2-3p	3	1.68E−09
hsa-miR-505-3p.2	0	8.96E−34	hsa-miR-892c-3p/hsa-miR-452-5p	1	8.81E−14	hsa-miR-376c-3p	4	9.51E−09
hsa-miR-1298-5p	0	1.88E−31	hsa-miR-4424	4	4.26E−12	hsa-miR-1249-3p	0	4E−08
hsa-miR-556-5p	0	3.4E−28	hsa-miR-339-5p	3	8.51E−11	hsa-miR-3605-3p	3	1.2E−07
hsa-miR-325-3p	3	5.95E−25	hsa-miR-944	0	8.51E−11	hsa-miR-143-3p	3	2.06E−07
hsa-miR-495-3p	0	1.05E−23	hsa-miR-345-5p	1	2.03E−10	hsa-miR-514b-5p/hsa-miR-513c-5p	1	6.57E−07
hsa-miR-1278	1	1.05E−23	hsa-miR-518c-3p	0	8.89E−10	hsa-miR-187-3p	3	1.39E−06
hsa-miR-651-5p	0	1.46E−22	hsa-miR-154-5p	2	3.17E−09	hsa-miR-625-3p	1	3.06E−06
hsa-miR-323b-3p	0	6.39E−18	hsa-miR-1251-5p	1	3.47E−09	hsa-miR-1306-5p	2	5.83E−06
hsa-miR-421	0	1.16E−17	hsa-miR-599	1	3.47E−09	hsa-miR-873-5p.1	3	7.82E−06
hsa-miR-876-5p	0	1.43E−17	hsa-miR-216a-5p	1	1.21E−08	hsa-miR-155-5p	2	7.82E−06
hsa-miR-150-5p	1	1.49E−17	hsa-miR-542-3p	1	1.35E−08	hsa-miR-942-5p	3	1.40E−05
hsa-miR-487b-3p	0	4.13E−17	hsa-miR-410-3p	1	1.42E−08	hsa-miR-382-3p	2	1.40E−05
hsa-miR-542-3p	1	1.01E−16	hsa-miR-524-5p	0	1.90E−08	hsa-miR-545-5p	1	1.61E−05
hsa-miR-380-3p	0	8.57E−16	hsa-miR-7151-5p	0	1.92E−07	hsa-miR-4428	1	2.43E−05

#### Experimentally supported interactions

PanomiR detected 202, 104 and 1 miRNA regulators in clusters A, B and C, respectively (FDR < 10^−5^, Tables 3 and S5). These included known liver cancer-associated miRNAs with consistent modes of action with their targeted pathway clusters. Cluster A was targeted by miR-525-3p, miR-1307, miR-631 and miR-663a—these miRNAs have been previously shown to have a role in tumor migration and invasion [65–68]. Cluster B was targeted by miRNAs with established roles in regulating cell cycle in liver cancer including miR-107, miR-124-3p and miR-103a-3p. For example, miR-107 is a P53-associated regulator of cell cycle and proliferation, elevated in early stage liver cancer [69–72]; miR-124-3p is a tumor suppressor that regulates proliferation and invasion in liver cancer by inducing G1-phase cell-cycle arrest [73, 74]; and miR-103a-3p is a promoter of proliferation that is highly dysregulated in liver cancer [75]. In cluster C, we found miR-410-3p as a central regulator of the relevant module. This miRNA has been shown to be a circulating biomarker of distant metastasis into the lung and the liver [76, 77]; it also regulates adenomas via signaling pathways such as MAPK, PTEN/AKT and STAT [78, 79]. In cluster C, we also found a significant targeting role for miR-552-3p, which has been associated with liver cancer and regulates various hallmarks of cancer [80]. Supplementary material provides an examination of the relationship between PanomiR miRNAs with differentially expressed miRNAs in liver cancer. While we did not find a significant association between prioritization by PanomiR and differential expression, PanomiR attributes distinct DE miRNAs to distinct groups of pathway-targeting events—providing a knowledge-driven approach for functional characterization of data-driven disease miRNAs (Tables S7–S9). Our results establish that PanomiR successfully detects key regulating liver-cancer miRNAs and their downstream differentially regulated pathways based on gene expression data.

#### Predicted miRNA–mRNA interactions

We also assessed PanomiR’s miRNA prioritization using predicted miRNA–mRNA interactions from the TargetScan database [44]. Although PanomiR detected multiple liver cancer-associated miRNAs from predicted interactions, the set of prioritized miRNAs was different than that of experimentally supported interactions (Tables 4 and S6). For example, PanomiR prioritized miR-299-3p in cluster C, a regulator of IL and STAT signaling pathways in liver cells, which have several associated annotated pathways in cluster C [81]. Our results suggest that predicted and experimentally validated miRNA interactions databases produce complementary results, and both should be considered for the downstream analysis of transcriptomic data.

### Parameter sensitivity analysis

We evaluated the sensitivity of PanomiR to input parameters and algorithmic choices. Our results show that PanomiR produces consistent results across varying input sizes, clustering algorithms and cut-offs for selecting predicted miRNA–mRNA interactions. We also show that PanomiR’s estimated miRNA prioritization *P*-values are robust and not driven by individual pathways.

#### Sensitivity to the number of input pathways

We asked whether the number of significant pathways selected in the module detection step (Figure 1D) affected the organization of pathway clusters. We iteratively performed Louvain clustering on the top pathways (ranging from 150 to 450 pathways with 50 increments) and assessed the Jaccard similarity between the top three clusters (Supplementary Material). Our results showed a strong conservation of module composition across varying parameter choices with a high-level one-to-one correspondence between the top three clusters in all iterations (Figure S2).

#### Assessment of different clustering algorithms

We investigated whether the choice of clustering algorithm affected the pathway module composition. PanomiR supports a range of clustering algorithms using the igraph package [55]. We evaluated the Jaccard similarity between the top three clusters within the top 200 differentially regulated pathways identified by Louvain, edge-betweenness, Infomap and fast-greedy clustering algorithms [82–85]. The fast-greedy and Louvain algorithms distributed pathways across three clusters, while the edge-betweenness and Infomap methods distributed the pathways mainly into two clusters (Figure S3A). Our results showed a strong one-to-one correspondence between the clusters generated by fast-greedy and Louvain as well as between the clusters generated by edge-betweenness and Infomap algorithms (Figure S3B). The results suggest an overall high-level stability of pathway modules using varying algorithms.

#### Stability of PanomiR’s miRNA prioritization *P*-values

We assessed the robustness and sensitivity of PanomiR’s miRNA prioritization by analyzing if the *P*-values of miRNA targeting were driven by individual pathways. We used a jackknifing strategy to recalculate PanomiR’s *P*-values in the clusters of differentially regulated pathways by leaving out one pathway at a time (Supplementary Material). The jackknifed *P*-values perfectly correlated with the original PanomiR *P*-values (Spearman’s rho = 1, Figure S4). This result shows that PanomiR’s miRNA prioritization depends on the collective targeting of pathways by each miRNA and is undriven by individual pathways. Additionally, we compared miRNA *P*-values estimated using the Gaussian method with those derived from bootstrapping. We found a significant correlation (Spearman’s rho = 0.9997, Figure S5), which demonstrates the stability of PanomiR’s estimated *P*-values.

#### Parameter sensitivity in using predicted miRNA–mRNA interactions

We assessed PanomiR’s miRNA prioritization using varying parameters for the selection of predicted miRNA–mRNA interactions in the TargetScan database (details in Supplementary Material). We deployed four strategies to generate lists of predicted miRNA targets. These strategies either used a combination of conserved miRNA families along with prediction scores or solely used various cut-offs for miRNA-mRNA binding prediction scores (Table S10). Using rank-based correlation analysis and hierarchical clustering, we show that PanomiR-derived miRNA ranking for targeting the three clusters of differentially regulated pathways is consistent and positively correlated across various selections of predicted miRNA–mRNA interactions (Figure S6).

### Comparison of PanomiR with existing miRNA–pathway analysis tools

We compared PanomiR with widely used miRNA–pathway analysis tools including DIANA-miRPath v4.0, MIENTURNET and MITHRIL v2.1 [22, 24, 33]. Many state-of-the-art tools use extensions of the over-representation analysis to infer miRNA–pathway interactions. To our knowledge, DIANA-miRPath is the only existing tool that directly assesses multi-pathway targeting by miRNAs. DIANA-miRPath uses Fisher’s aggregation on miRNA-pathway over-representation *P*-values (referred to as ‘term-centric’ analysis). However, in contrast to PanomiR that aims to prioritize transcriptional programs spanning multiple pathways controlled by specific miRNAs, miRPath’s aggregation statistic returns whether the selected miRNA significantly targets at least one of the identified pathways [33]. Other tools, including miRPathDB v2.0, can determine how many pathways are enriched in gene targets of a queried miRNA, which we refer to as ‘miRNA hits’. Unlike PanomiR, most tools (including DIANA-miRPath, miRPathDB and miTalos) do not provide an objective selection of input pathways and leave the input to the users. We compared our system with other tools using both the original software (where functional and applicable) and their adaptations within PanomiR’s system.

For a fair comparison using the same background of genes and pathways, we assessed two adaptations of existing miRNA–pathway analysis approaches in the PanomiR’s ecosystem: ‘miRNA hits’ and ‘*P*-value aggregation’. Specifically, we extended the enrichment analysis to a group (cluster) of pathways by interrogating the number of pathways within a given cluster that were significantly enriched for targets of a miRNA (miRNA hits). This ‘miRNA hits’ approach is similar to the functionalities within miRPathDB v2.0 and miTalos [23, 27]. For example, if the targets of a miRNA are significantly enriched in five pathways within a group of pathways, the miRNA receives a targeting (hit) score of 5. For the ‘*P*-value aggregation’ approach, we used Fisher’s method to obtain one single *P*-value that combines enrichment *P*-values of a miRNA within all pathways in a cluster. This approach has been previously implemented in DIANA-miRPath v4.0 for multi-pathway targeting. We also analyzed Stouffer’s *P*-value aggregation method within clusters to combine miRNA–pathway enrichment *P*-values as an alternative of Fisher’s method.

#### Prioritization of disease-associated miRNAs

PanomiR successfully detected liver cancer-associated miRNAs that were not prioritized by enrichment analysis (miRNA hits) and *P*-value aggregation approaches (Tables 3 and S5). When using experimentally supported miRNA–mRNA interactions, enrichment analysis of cluster A revealed miR-525-3p as enriched in only 1 and miR-1307-5p in none out of 65 pathways (Table 3). Fisher’s aggregation-adjusted *P*-values for miR-525-3p and miR-1307-5p were 1.3 × 10^−9^ and 9.15 × 10^−1^, respectively (Table S5). When using predicted miRNA–mRNA interactions, PanomiR detected several miRNAs that were not detected by the number hits in the enrichment analysis (Table 4). It is worth noting that the enrichment tests (Tables 3 and 4) used a relaxed threshold (FDR < 0.25) to allow for more sensitive detection. Using a conservative cut-off (e.g., FDR < 0.05) would have retained an even lower detection rate of miRNAs. The results suggest that (a) PanomiR can detect liver cancer-associated miRNAs that are not detectable by simple enrichment tests or current miRNA pathway approaches and (b) a subset of critical liver cancer miRNAs can be detected only by analyzing a group of pathways in the form of coordinated programs and not by examining individual pathways.

#### Bias of existing methods in prioritizing miRNAs with more targeted genes

**Figure 5:**
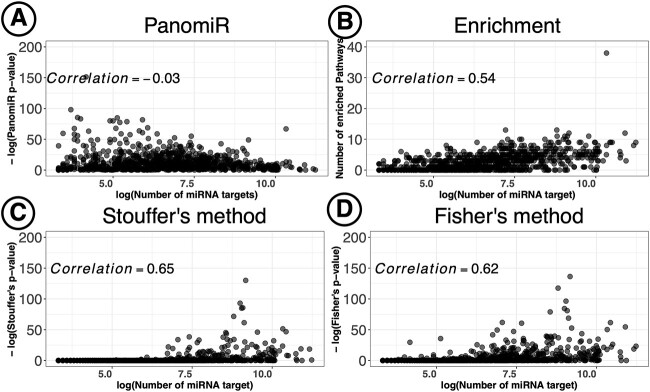
Unbiased prioritization of miRNAs by PanomiR. PanomiR prioritizes miRNAs with either a small or large number of annotated targets. In contrast, enrichment-based miRNA-prioritization methods are biased toward prioritization of miRNAs with larger numbers of gene targets. The figure displays correlation analysis of miRNA-prioritization rankings with the number of gene targets in Cluster A of the liver cancer data set. Each point represents a miRNA annotated in the TarBase database. (**A**) Spearman correlation analysis did not find a significant association between the number of targets and the prioritization ranking of miRNAs by PanomiR (correlation −0.03). (**B**) The number of enriched pathways for a miRNA significantly correlated with its number of gene targets. We also observed a significant correlation between the number of a miRNA’s targets and its prioritization ranking based on (**C**) Stouffer’s method and (**D**) Fisher’s method for aggregation of enrichment *P*-value. *X*-axes denote the log number of gene targets of miRNAs based on experimentally validated miRNA–mRNA interactions from the TarBase database. The *y*-axis in (B) represents the number of significantly enriched pathways (Adjusted *P*-value < 0.25, Table 3).

One-to-one miRNA-pathway enrichment analyses have been shown to be biased toward detecting miRNAs with a larger number of targets [56]. For the standard multi-pathway strategies discussed above, we examined the relationship of the number of targets of a miRNA with its prioritization ranking (Tables 4 and S5). The enrichment score (hits) ranking of miRNAs significantly correlated with their number of gene targets. Stouffer’s and Fisher’s *P*-value aggregation methods also showed a strong correlation between the number of miRNA targets and the *P*-value ranking in cluster A of the TCGA data. These results suggest that current commonly available approaches for multi-pathway targeting are biased toward prioritizing miRNAs with more targets. In comparison, PanomiR did not show a significant correlation between ranking of a miRNA (based on *P*-value) and the number of its targets (Figure 5), suggesting its ability to prioritize miRNAs irrespective of the number of their gene targets.

In addition to our local implementation of miRPath v4.0 (Fisher’s aggregation, Figure 5), we directly compared our results with the most recent online version of miRPath v4.0 using its ‘Term-centric’ analysis (Supplementary Material) [33]. miRPath’s portal only allowed 20 input pathways at each query. Thus, we manually queried the top 20 pathways in cluster A of the TCGA liver cancer data. miRPath v4.0 identified 193 miRNA targeting the top 20 pathways in cluster A (Table S1). Concordant with our Fisher’s aggregation implementation results, miRPath showed a significant bias toward prioritization of miRNAs with a higher number of gene targets (Spearman’s rho = 0.48, Figure S7).

#### Other comparisons

We compared PanomiR with MIENTURNET (a network-based prioritization method of regulating miRNAs) by querying the list of DE genes in the online portal [22]. MIENTURNET identified three significant miRNAs targeting events (FDR < 0.05) (Table S12): miR-192-5p, miR-215-5p and miR-193-5p. MIENTURNET does not directly determine pathway deregulation events and infers miRNA targeting using gene interactions networks. The online portal detected functional pathway enrichment of miR-192-5p and miR-193b-5p using the interaction network of the input list genes. The MIENTURNET’s enrichment results predominantly included terms related to cell cycle, overlapping with cluster B of PanomiR. PanomiR determined miR-192-5p as a top miRNA targeting the differentially regulated pathways in cluster B. We also compared PanomiR with MITHRIL, which detects pathway dysregulation using DE genes or DE miRNAs. MITHRIL detected nine significantly deregulated KEGG pathways (FDR < 0.01), mainly related to metabolic pathways. Among these, ‘Drug metabolism – cytochrome P450’, ‘Retinol metabolism’ and ‘Linoleic acid metabolism’ were represented in cluster C of PanomiR (Tables S4 and S13). Our results demonstrate that when compared against existing methods, PanomiR provides unique functionality, high sensitivity and results that are not affected by commonly observed biases.

## DISCUSSION

PanomiR is a framework to determine miRNA regulation of multiple coordinately regulated pathways. Most of the existing tools for miRNA–pathway analysis are focused on one-to-one miRNA–pathway relationships and lack the ability to infer relationships between miRNAs and their groups of co-regulated pathways. Approaches to address this problem have used standard enrichment analysis as a basis for interrogation of multi-pathway targeting. However, this approach does not take into account the interaction between pathways and their relative expression dynamics [86]. Alternative gene-network-centric miRNA-analysis approaches, such as MIENTURNET, do not directly examine the interactions of miRNAs with known pathways. In network-based tools, disease pathways are only inferred from gene-network modules [22, 87–89]. PanomiR addresses these challenges by deconvolving gene expression into coordinate groups of differentially regulated pathways; measuring the extent to which miRNAs target these groups. Applied to a case study of liver hepatocellular carcinoma, PanomiR captured broad-scale characteristics of cancers such as dysregulation of transcription, cellular replication and signaling (Figure 3). These groups, composed of differentially regulated pathways, represented coherent higher-order functional units that recapitulated specific, yet central, disease mechanisms.

The use of pathway activity profiles is a key component of PanomiR. It sensitively detects differentially regulated pathways and provides granular definition of coordinate functional groups (Figures 2 and 3, Table 2). PanomiR detected critical known liver cancer pathways, even though there were few differentially expressed genes associated with them (Table 2). Pathway activity profiles in PanomiR determine the up- and downregulation of pathways. The activity profiles are directly comparable and translatable across different experiments, which makes it possible to leverage co-expression of pathways to detect disease-specific functional dynamics and themes across data sets, platforms and species [41].

PanomiR’s multi-pathway approach provided unbiased detection of miRNA regulatory events in liver cancer that was not detectable by conventional analyses (Figure 4, Table 3). By using 113 K high-confidence predicted miRNA interactions (TargetScan) and more than 500 K experimentally supported (TarBase) miRNA targets, PanomiR discovered informative and complementary miRNA regulatory events (Tables 3 and 4). The prioritized miRNAs identified by PanomiR have distinct roles in the disease and target pathways. The results also show that PanomiR can identify key miRNAs that target groups of pathways even with only a few targeted genes within individual pathways.

## CONCLUSION

PanomiR provides an advancement over the current practice of studying static, isolated miRNA–pathway interactions. It is the first systems biology framework to study multiple differentially regulated pathways, their co-activity and their regulating miRNAs. The model achieves its broad-scale inference by accounting for co-expression of pathways and disease-specific expression dynamics to identify miRNA-regulatory events.

PanomiR provides multiple functionalities that are not available through established miRNA–pathway analysis tools. These include pathway dysregulation analysis, identification of groups of co-expressed pathways for miRNA prioritization and granular assignment of disease-associated miRNAs to specific groups of disease pathways. These features enable PanomiR to produce unbiased miRNA prioritization results, which, in turn, sensitively determine key disease miRNAs and their targeted pathways even when there are only few known gene targets or significant differentially expressed genes.

Limitations of the PanomiR approach include (1) PanomiR detects one-to-many miRNA–pathway relationships and does not provide the analysis of many-to-one relationships; (2) PanomiR’s infers miRNA from gene expression data. In addition, PanomiR does not provide co-expression analysis between pathways and miRNAs. Additional cross-examination with miRNA expression data may be necessary to make the results more actionable, representing a potential area for future expansion; (3) PanomiR primarily works with bulk RNA-Seq data and is not designed for single-cell/spatial transcriptomic data sets. For users who are interested in single-cell/spatial transcriptomic analysis of miRNA–pathway relationships, we suggest adapting a pseudo-bulk transformation prior to using PanomiR; and (4) the complete landscape of miRNA–mRNA binding relationships is unknown. This gap can drive discrepancy in miRNA prioritization based on different background data sets of miRNA–mRNA interactions (examples in Tables 3 and 4). In order to mitigate this gap, we have made PanomiR adaptable to varying miRNA–mRNA interaction databases, pathway gene sets and gene expression data sets to facilitate user-specific study designs and research questions. PanomiR is available to the community as an open-source R/Bioconductor package.

**Key Points**

- Characterizing the roles of miRNAs in health and disease requires charting of coordinate co-regulation across heterogeneous molecular cascades and pathways. PanomiR is the first-of-its-kind method aiming to capture the effects of miRNAs in coordinating multi-pathway transcriptional programs.
- PanomiR enables the detection of differential pathway regulation events between two groups. It uses pathway activity profiling to identify phenotype/group-associated transcriptional programs using a co-expression network of disease-associated pathways.
- PanomiR achieves sensitive and unbiased prioritization of disease-regulating miRNAs using statistical tests for analyzing one-to-many miRNA–pathway relationships within groups of coordinate disease functions.
- PanomiR is available as a user-friendly, open-source software package. It enables using a diverse range of clustering algorithms, background pathways and miRNA–mRNA interaction databases.

## Supplementary Material

supplementary_material_2023_10_30_final_bbad418

supplementary_tables_bbad418

## Data Availability

PanomiR is available as an open source R/Bioconductor package https://bioconductor.org/packages/PanomiR. The development version of PanomiR can be accessed via the following GitHub repository: https://github.com/pouryany/PanomiR. Additional scripts, data sets and analyses, specific to this manuscript, are available via https://github.com/pouryany/PanomiR_paper.

## References

[1] Friedman RC , Farh KK-H, Burge CB, et al. Most mammalian mRNAs are conserved targets of microRNAs. Genome Res 2009;19:92–105. 18955434 10.1101/gr.082701.108 PMC2612969

[2] Slack FJ , Chinnaiyan AM. The role of non-coding RNAs in oncology. Cell 2019;179:1033–55. 31730848 10.1016/j.cell.2019.10.017 PMC7347159

[3] Cai Y , Yu X, Hu S, et al. A brief review on the mechanisms of miRNA regulation. Genomics Proteomics Bioinformatics 2009;7:147–54. 20172487 10.1016/S1672-0229(08)60044-3 PMC5054406

[4] Wilk G , Braun R. Integrative analysis reveals disrupted pathways regulated by microRNAs in cancer. Nucleic Acids Res 2018;46:1089–101. 29294105 10.1093/nar/gkx1250 PMC5814839

[5] Cui Q , Yu Z, Purisima EO, et al. Principles of microRNA regulation of a human cellular signaling network. Mol Syst Biol 2006;2:46. 16969338 10.1038/msb4100089 PMC1681519

[6] Artmann S , Jung K, Bleckmann A, et al. Detection of simultaneous group effects in microRNA expression and related target gene sets. PloS One 2012;7:e38365. 22723856 10.1371/journal.pone.0038365 PMC3378551

[7] Werfel S , Leierseder S, Ruprecht B, et al. Preferential microRNA targeting revealed by in vivo competitive binding and differential Argonaute immunoprecipitation. Nucleic Acids Res 2017;45:10218–28. 28973447 10.1093/nar/gkx640 PMC5622317

[8] Peláez N , Carthew RW. Biological robustness and the role of microRNAs: a network perspective. Curr Top Dev Biol 2012;99:237–55. 22365741 10.1016/B978-0-12-387038-4.00009-4 PMC3746555

[9] Kehl T , Backes C, Kern F, et al. About miRNAs, miRNA seeds, target genes and target pathways. Oncotarget 2017;8:107167–75. 29291020 10.18632/oncotarget.22363 PMC5739805

[10] Pasquinelli AE . MicroRNAs and their targets: recognition, regulation and an emerging reciprocal relationship. Nat Rev Genet 2012;13:271–82. 22411466 10.1038/nrg3162

[11] Kozomara A , Griffiths-Jones S. miRBase: annotating high confidence microRNAs using deep sequencing data. Nucleic Acids Res 2014;42:D68–73. 24275495 10.1093/nar/gkt1181 PMC3965103

[12] Mishima Y . Widespread roles of microRNAs during zebrafish development and beyond. Dev Growth Differ 2012;54:55–65. 22150108 10.1111/j.1440-169X.2011.01306.x

[13] Hashimoto N , Tanaka T. Role of miRNAs in the pathogenesis and susceptibility of diabetes mellitus. J Hum Genet 2017;62:141–50. 27928162 10.1038/jhg.2016.150

[14] Deng X , Su Y, Wu H, et al. The role of microRNAs in autoimmune diseases with skin involvement. Scand J Immunol 2015;81:153–65. 25430682 10.1111/sji.12261

[15] Reinhart BJ , Slack FJ, Basson M, et al. The 21-nucleotide let-7 RNA regulates developmental timing in *Caenorhabditis elegans*. Nature 2000;403:901–6. 10706289 10.1038/35002607

[16] Esquela-Kerscher A , Slack FJ. Oncomirs - microRNAs with a role in cancer. Nat Rev Cancer 2006;6:259–69. 16557279 10.1038/nrc1840

[17] Lima RT , Busacca S, Almeida GM, et al. MicroRNA regulation of core apoptosis pathways in cancer. Eur J Cancer 2011;47:163–74. 21145728 10.1016/j.ejca.2010.11.005

[18] Sumazin P , Yang X, Chiu H-S, et al. An extensive microRNA-mediated network of RNA-RNA interactions regulates established oncogenic pathways in glioblastoma. Cell 2011;147:370–81. 22000015 10.1016/j.cell.2011.09.041 PMC3214599

[19] Gascon E , Gao F-B. Cause or effect: Misregulation of microRNA pathways in neurodegeneration. Front Neurosci 2012;6:48. 22509148 10.3389/fnins.2012.00048 PMC3321503

[20] Rao X , Di Leva G, Li M, et al. MicroRNA-221/222 confers breast cancer fulvestrant resistance by regulating multiple signaling pathways. Oncogene 2011;30:1082–97. 21057537 10.1038/onc.2010.487 PMC3342929

[21] Vlachos IS , Zagganas K, Paraskevopoulou MD, et al. DIANA-miRPath v3.0: deciphering microRNA function with experimental support. Nucleic Acids Res 2015;43:W460–6. 25977294 10.1093/nar/gkv403 PMC4489228

[22] Licursi V , Conte F, Fiscon G, et al. MIENTURNET: an interactive web tool for microRNA-target enrichment and network-based analysis. BMC Bioinformatics 2019;20:545. 31684860 10.1186/s12859-019-3105-x PMC6829817

[23] Kehl T , Kern F, Backes C, et al. miRPathDB 2.0: a novel release of the miRNA pathway dictionary database. Nucleic Acids Res 2020;48:D142–7. 31691816 10.1093/nar/gkz1022 PMC7145528

[24] Alaimo S , Giugno R, Acunzo M, et al. Post-transcriptional knowledge in pathway analysis increases the accuracy of phenotypes classification. Oncotarget 2016;7:54572–82. 27275538 10.18632/oncotarget.9788 PMC5342365

[25] Hsu JB-K , Chiu C-M, Hsu S-D, et al. miRTar: an integrated system for identifying miRNA-target interactions in human. BMC Bioinformatics 2011;12:300. 21791068 10.1186/1471-2105-12-300 PMC3162936

[26] Zagganas K , Vergoulis T, Paraskevopoulou MD, et al. BUFET: boosting the unbiased miRNA functional enrichment analysis using bitsets. BMC Bioinformatics 2017;18:399. 28874117 10.1186/s12859-017-1812-8 PMC5585958

[27] Preusse M , Theis FJ, Mueller NS. MiTALOS v2: Analyzing tissue specific microRNA function. PloS One 2016;11:e0151771. 26998997 10.1371/journal.pone.0151771 PMC4801359

[28] Chen L , Heikkinen L, Wang C, et al. miRToolsGallery: a tag-based and rankable microRNA bioinformatics resources database portal. Database 2018;2018:bay004. 10.1093/database/bay004 PMC5819725 29688355

[29] Chen L , Heikkinen L, Wang C, et al. Trends in the development of miRNA bioinformatics tools. Brief Bioinform 2019;20:1836–52. 29982332 10.1093/bib/bby054 PMC7414524

[30] Backes C , Khaleeq QT, Meese E, et al. miEAA: microRNA enrichment analysis and annotation. Nucleic Acids Res 2016;44:W110–6. 27131362 10.1093/nar/gkw345 PMC4987907

[31] Steinfeld I , Navon R, Ach R, et al. miRNA target enrichment analysis reveals directly active miRNAs in health and disease. Nucleic Acids Res 2013;41:e45. 23209027 10.1093/nar/gks1142 PMC3561970

[32] Li J , Han X, Wan Y, et al. TAM 2.0: tool for MicroRNA set analysis. Nucleic Acids Res 2018;46:W180–5. 29878154 10.1093/nar/gky509 PMC6031048

[33] Tastsoglou S , Skoufos G, Miliotis M, et al. DIANA-miRPath v4.0: expanding target-based miRNA functional analysis in cell-type and tissue contexts. Nucleic Acids Res 2023;51:W154–9. 37260078 10.1093/nar/gkad431 PMC10320185

[34] Braun R , Cope L, Parmigiani G. Identifying differential correlation in gene/pathway combinations. BMC Bioinformatics 2008;9:488. 19017408 10.1186/1471-2105-9-488 PMC2613418

[35] Khatri P , Sirota M, Butte AJ. Ten years of pathway analysis: current approaches and outstanding challenges. PLoS Comput Biol 2012;8:e1002375. 22383865 10.1371/journal.pcbi.1002375 PMC3285573

[36] Rhodes DR , Kalyana-Sundaram S, Tomlins SA, et al. Molecular concepts analysis links tumors, pathways, mechanisms, and drugs. Neoplasia 2007;9:443–54. 17534450 10.1593/neo.07292 PMC1877973

[37] Merico D , Isserlin R, Stueker O, et al. Enrichment map: a network-based method for gene-set enrichment visualization and interpretation. PloS One 2010;5:e13984. 21085593 10.1371/journal.pone.0013984 PMC2981572

[38] Huang Y , Li S. Detection of characteristic sub pathway network for angiogenesis based on the comprehensive pathway network. BMC Bioinformatics 2010;11(Suppl 1):S32. 20122205 10.1186/1471-2105-11-S1-S32 PMC3009504

[39] Li Y , Agarwal P, Rajagopalan D. A global pathway crosstalk network. Bioinformatics 2008;24:1442–7. 18434343 10.1093/bioinformatics/btn200

[40] Karlebach G , Shamir R. Modelling and analysis of gene regulatory networks. Nat Rev Mol Cell Biol 2008;9:770–80. 18797474 10.1038/nrm2503

[41] Altschuler GM , Hofmann O, Kalatskaya I, et al. Pathprinting: an integrative approach to understand the functional basis of disease. Genome Med 2013;5:68. 23890051 10.1186/gm472 PMC3971351

[42] Joachim RB , Altschuler GM, Hutchinson JN, et al. The relative resistance of children to sepsis mortality: from pathways to drug candidates. Mol Syst Biol 2018;14:e7998. 29773677 10.15252/msb.20177998 PMC5974511

[43] Pita-Juárez Y , Altschuler G, Kariotis S, et al. The pathway coexpression network: revealing pathway relationships. PLoS Comput Biol 2018;14:e1006042. 29554099 10.1371/journal.pcbi.1006042 PMC5875878

[44] Agarwal V , Bell GW, Nam J-W, et al. Predicting effective microRNA target sites in mammalian mRNAs. Elife 2015;4:e05005. 10.7554/eLife.05005 PMC4532895 26267216

[45] Karagkouni D , Paraskevopoulou MD, Chatzopoulos S, et al. DIANA-TarBase v8: a decade-long collection of experimentally supported miRNA-gene interactions. Nucleic Acids Res 2018;46:D239–45. 29156006 10.1093/nar/gkx1141 PMC5753203

[46] Liberzon A , Subramanian A, Pinchback R, et al. Molecular signatures database (MSigDB) 3.0. Bioinformatics 2011;27:1739–40. 21546393 10.1093/bioinformatics/btr260 PMC3106198

[47] Fan J , Salathia N, Liu R, et al. Characterizing transcriptional heterogeneity through pathway and gene set overdispersion analysis. Nat Methods 2016;13:241–4. 26780092 10.1038/nmeth.3734 PMC4772672

[48] Tomfohr J , Lu J, Kepler TB. Pathway level analysis of gene expression using singular value decomposition. BMC Bioinformatics 2005;6:225. 16156896 10.1186/1471-2105-6-225 PMC1261155

[49] Subramanian A , Tamayo P, Mootha VK, et al. Gene set enrichment analysis: a knowledge-based approach for interpreting genome-wide expression profiles. Proc Natl Acad Sci U S A 2005;102:15545–50. 16199517 10.1073/pnas.0506580102 PMC1239896

[50] Kanehisa M , Furumichi M, Tanabe M, et al. KEGG: new perspectives on genomes, pathways, diseases and drugs. Nucleic Acids Res 2017;45:D353–61. 27899662 10.1093/nar/gkw1092 PMC5210567

[51] Jassal B , Matthews L, Viteri G, et al. The reactome pathway knowledgebase. Nucleic Acids Res 2020;48:D498–503. 31691815 10.1093/nar/gkz1031 PMC7145712

[52] Ritchie ME , Phipson B, Wu D, et al. Limma powers differential expression analyses for RNA-sequencing and microarray studies. Nucleic Acids Res 2015;43:e47. 25605792 10.1093/nar/gkv007 PMC4402510

[53] Benjamini Y , Hochberg Y. Controlling the false discovery rate: a practical and powerful approach to multiple testing. J R Stat Soc 1995;57:289–300.

[54] McCall MN , Jaffee HA, Zelisko SJ, et al. The Gene Expression Barcode 3.0: improved data processing and mining tools. Nucleic Acids Res 2014;42:D938–43. 24271388 10.1093/nar/gkt1204 PMC3965035

[55] Csardi G , Nepusz T. The igraph software package for complex network research. Int J Complex Syst 2006;1695:1–9.

[56] Bleazard T , Lamb JA, Griffiths-Jones S. Bias in microRNA functional enrichment analysis. Bioinformatics 2015;31:1592–8. 25609791 10.1093/bioinformatics/btv023 PMC4426843

[57] Godard P , van Eyll J. Pathway analysis from lists of microRNAs: common pitfalls and alternative strategy. Nucleic Acids Res 2015;43:3490–7. 25800743 10.1093/nar/gkv249 PMC4402548

[58] Cancer Genome Atlas Research Network . Comprehensive and integrative genomic characterization of hepatocellular carcinoma. Cell 2017;169:1327–1341.e23. 28622513 10.1016/j.cell.2017.05.046 PMC5680778

[59] Mounir M , Lucchetta M, Silva TC, et al. New functionalities in the TCGAbiolinks package for the study and integration of cancer data from GDC and GTEx. PLoS Comput Biol 2019;15:e1006701. 30835723 10.1371/journal.pcbi.1006701 PMC6420023

[60] Ito Y , Takeda T, Sakon M, et al. Expression and clinical significance of erb-B receptor family in hepatocellular carcinoma. Br J Cancer 2001;84:1377–83. 11355950 10.1054/bjoc.2000.1580 PMC2363640

[61] Liu Y , Song L, Ni H, et al. ERBB4 acts as a suppressor in the development of hepatocellular carcinoma. Carcinogenesis 2017;38:465–73. 28334319 10.1093/carcin/bgx017

[62] Jiang J , Nilsson-Ehle P, Xu N. Influence of liver cancer on lipid and lipoprotein metabolism. Lipids Health Dis 2006;5:4. 16515689 10.1186/1476-511X-5-4 PMC1420303

[63] Jiang J-T , Xu N, Wu C-P. Metabolism of high density lipoproteins in liver cancer. World J Gastroenterol 2007;13:3159–63. 17589892 10.3748/wjg.v13.i23.3159 PMC4436599

[64] Wang W , Smits R, Hao H, et al. Wnt/β-catenin signaling in liver cancers. Cancers 2019;11:926. 31269694 10.3390/cancers11070926 PMC6679127

[65] Pang F , Zha R, Zhao Y, et al. MiR-525-3p enhances the migration and invasion of liver cancer cells by downregulating ZNF395. PloS One 2014;9:e90867. 24599008 10.1371/journal.pone.0090867 PMC3944804

[66] Eun JW , Seo CW, Baek GO, et al. Circulating exosomal MicroRNA-1307-5p as a predictor for metastasis in patients with hepatocellular carcinoma. Cancers 2020;12:3819. 33352935 10.3390/cancers12123819 PMC7766543

[67] Chen B , Liao Z, Qi Y, et al. MiR-631 inhibits intrahepatic metastasis of hepatocellular carcinoma by targeting PTPRE. Front Oncol 2020;10:565266. 33344226 10.3389/fonc.2020.565266 PMC7746836

[68] Zhang C , Chen B, Jiao A, et al. miR-663a inhibits tumor growth and invasion by regulating TGF-β1 in hepatocellular carcinoma. BMC Cancer 2018;18:1179. 30486878 10.1186/s12885-018-5016-z PMC6264054

[69] Böhlig L , Friedrich M, Engeland K. p53 activates the PANK1/miRNA-107 gene leading to downregulation of CDK6 and p130 cell cycle proteins. Nucleic Acids Res 2011;39:440–53. 20833636 10.1093/nar/gkq796 PMC3025554

[70] Zhang J-J , Wang C-Y, Hua L, et al. miR-107 promotes hepatocellular carcinoma cell proliferation by targeting Axin2. Int J Clin Exp Pathol 2015;8:5168–74. 26191213 PMC4503085

[71] Takahashi Y , Forrest ARR, Maeno E, et al. MiR-107 and MiR-185 can induce cell cycle arrest in human non small cell lung cancer cell lines. PloS One 2009;4:e6677. 19688090 10.1371/journal.pone.0006677 PMC2722734

[72] Loosen SH , Castoldi M, Jördens MS, et al. Serum levels of circulating microRNA-107 are elevated in patients with early-stage HCC. PloS One 2021;16:e0247917. 33711036 10.1371/journal.pone.0247917 PMC7954311

[73] Lang Q , Ling C. MiR-124 suppresses cell proliferation in hepatocellular carcinoma by targeting PIK3CA. Biochem Biophys Res Commun 2012;426:247–52. 22940133 10.1016/j.bbrc.2012.08.075

[74] Callegari E , Gramantieri L, Domenicali M, et al. MicroRNAs in liver cancer: a model for investigating pathogenesis and novel therapeutic approaches. Cell Death Differ 2015;22:46–57. 25190143 10.1038/cdd.2014.136 PMC4262781

[75] Liu Y , Zhang Y, Xiao B, et al. MiR-103a promotes tumour growth and influences glucose metabolism in hepatocellular carcinoma. Cell Death Dis 2021;12:618. 34131101 10.1038/s41419-021-03905-3 PMC8206076

[76] Wang Y , Fu J, Jiang M, et al. MiR-410 is overexpressed in liver and colorectal tumors and enhances tumor cell growth by silencing FHL1 via a direct/indirect mechanism. PloS One 2014;9:e108708. 25272045 10.1371/journal.pone.0108708 PMC4182719

[77] Liu X , Chu K-M. Exosomal miRNAs as circulating biomarkers for prediction of development of haematogenous metastasis after surgery for stage II/III gastric cancer. J Cell Mol Med 2020;24:6220–32. 32383554 10.1111/jcmm.15253 PMC7294143

[78] Wen R , Umeano AC, Essegian DJ, et al. Role of microRNA-410 in molecular oncology: a double edged sword. J Cell Biochem 2018;119:8737–42. 30086210 10.1002/jcb.27251

[79] Grzywa TM , Klicka K, Rak B, et al. Lineage-dependent role of miR-410-3p as oncomiR in gonadotroph and corticotroph pituitary adenomas or tumor suppressor miR in somatotroph adenomas via MAPK, PTEN/AKT, and STAT3 signaling pathways. Endocrine 2019;65:646–55. 31165412 10.1007/s12020-019-01960-7 PMC6717603

[80] Zou Y , Zhao X, Li Y, et al. miR-552: an important post-transcriptional regulator that affects human cancer. J Cancer 2020;11:6226–33. 33033505 10.7150/jca.46613 PMC7532495

[81] Servais FA , Kirchmeyer M, Hamdorf M, et al. Modulation of the IL-6-signaling pathway in liver cells by miRNAs targeting gp130, JAK1, and/or STAT3. Mol Ther Nucleic Acids 2019;16:419–33. 31026677 10.1016/j.omtn.2019.03.007 PMC6479786

[82] Newman MEJ , Girvan M. Finding and evaluating community structure in networks. Phys Rev E Stat Nonlin Soft Matter Phys 2004;69:026113. 14995526 10.1103/PhysRevE.69.026113

[83] Blondel VD , Guillaume J-L, Lambiotte R, et al. Fast unfolding of communities in large networks. J Stat Mech 2008;2008:P10008.

[84] Brandes U . A faster algorithm for betweenness centrality. J Math Sociol 2001;25:163–77.

[85] Rosvall M , Bergstrom CT. Maps of random walks on complex networks reveal community structure. Proc Natl Acad Sci U S A 2008;105:1118–23. 18216267 10.1073/pnas.0706851105 PMC2234100

[86] Vlachos IS , Kostoulas N, Vergoulis T, et al. DIANA miRPath v.2.0: investigating the combinatorial effect of microRNAs in pathways. Nucleic Acids Res 2012;40:W498–504. 22649059 10.1093/nar/gks494 PMC3394305

[87] Zeng X , Zhang X, Zou Q. Integrative approaches for predicting microRNA function and prioritizing disease-related microRNA using biological interaction networks. Brief Bioinform 2016;17:193–203. 26059461 10.1093/bib/bbv033

[88] Fan Y , Siklenka K, Arora SK, et al. miRNet - dissecting miRNA-target interactions and functional associations through network-based visual analysis. Nucleic Acids Res 2016;44:W135–41. 27105848 10.1093/nar/gkw288 PMC4987881

[89] Shi H , Xu J, Zhang G, et al. Walking the interactome to identify human miRNA-disease associations through the functional link between miRNA targets and disease genes. BMC Syst Biol 2013;7:101. 24103777 10.1186/1752-0509-7-101 PMC4124764

